# Cardiac arrhythmias during intense exercise in Thoroughbred racehorses: frequency and association with subsequent race performance

**DOI:** 10.1093/jvimsj/aalag044

**Published:** 2026-03-24

**Authors:** Emmanuelle van Erck-Westergren, Stephen O’Connor, Brian D Stewart, Guillaume Dubois, John Morton, Kenneth W Hinchcliff, Fe ter Woort

**Affiliations:** Equine Sports Medicine Practice, Waterloo, Belgium; Hong Kong Jockey Club, Department of Veterinary Clinical Services, Sha Tin Equine Hospital, Sha Tin, New Territories, Hong Kong; Hong Kong Jockey Club, Department of Veterinary Clinical Services, Sha Tin Equine Hospital, Sha Tin, New Territories, Hong Kong; Arioneo, LIM Group, Bordeaux, France; Jemora Pty Ltd, East Geelong, Victoria, Australia; Melbourne Veterinary School, University of Melbourne and Trinity College, Melbourne, Victoria, Australia; Equine Sports Medicine Practice, Waterloo, Belgium

**Keywords:** arrhythmia, cardiovascular, sports medicine, electrocardiography, equine, performance, racing

## Abstract

**Background:**

The frequency, characteristics, and relationship with performance of arrhythmias occurring during high-intensity exercise in Thoroughbred racehorses remain poorly understood.

**Hypothesis/Objectives:**

Describe the frequency, characteristics, and association with subsequent race performance of arrhythmias occurring during intense exercise.

**Animals:**

Seventy-one racehorses competing in races sanctioned by the Hong Kong Jockey Club.

**Methods:**

Risk-based case–control study. Single-lead ECGs were recorded during high-intensity trials using wearable devices. Ectopic depolarizations identified during warm-up, maximal exercise, and fast and slow recovery were characterized by frequency and morphology. Horses with ECG recordings obtained before a race were included. Race performance was classified as poor (finishing in the last 3 positions) or good (finishing in the first 3 positions). Associations between ECG variables and race performance were assessed using odds ratios.

**Results:**

A total of 405 ECGs were recorded within 21 days before 1 or more race starts (82 poor- and 142 good-performance starts). Arrhythmias were detected in 73% and 81% of ECGs preceding good and poor performances, respectively. Ectopic beats during maximal exercise occurred in 57% of ECGs before good and in 70% before poor performances. The odds of performing poorly increased with each additional ectopic beat (odds ratio [OR], 1.15; 95% confidence interval [CI], 1.04-1.28; *P* = .01), and horses with any ectopic beat were nearly twice as likely to underperform (OR, 1.8; 95% CI, 0.86-3.81; *P* = .02).

**Conclusions and clinical importance:**

Ectopic beats are common and adversely associated with athletic performance. Electrocardiographic monitoring during intense exertion might enable detection of clinically important arrhythmias.

## Introduction

Cardiac arrhythmias are common in equine athletes, particularly during intense exertion, with a spectrum of severity ranging from clinically benign to potentially life-threatening. Among the most clinically important arrhythmias are atrial fibrillation (AF) and complex ventricular arrhythmias, whereas isolated ectopic beats generally are considered of minimal consequence.^1-3^ Although isolated ectopic beats do not appear to affect athletic performance, complex tachydysrhythmias likely compromise performance and welfare, could pose substantial safety risks to both horse and rider, and potentially contribute to higher risk of sudden death during exercise.^1^ Furthermore, public perception of equestrian sports is increasingly shaped by concerns about deaths of horses during competitions, emphasizing the need to understand risk factors for exercise-associated sudden cardiac death (EASCD). An additional concern is that of unexplained and unexpected poor performance by otherwise healthy horses and the potential role of arrhythmias. These concerns prompt investigation of the potential role of proactive cardiovascular monitoring and management in identifying horses at risk of either outcome.

Exercise-associated sudden cardiac death is, after catastrophic musculoskeletal injury, the major cause of death of racehorses during exercise.^2^ Exercise-associated sudden cardiac death occurs more commonly during training than racing,^2^ suggesting that exercise intensity alone might not be the primary trigger. Similarly, although unexplained and sudden failure to perform to expectation during a race is commonly associated with musculoskeletal injury or exercise-induced pulmonary hemorrhage, for a proportion of horses the cause remains unknown. Transient exercise-associated arrhythmia is potentially an important cause of unexplained poor performance, based on small-scale treadmill or racetrack studies.^3-9^

Despite the clinical relevance of arrhythmias, no large-scale longitudinal studies have assessed the association of arrhythmias occurring during exercise with performance during racing. The unpredictability of EASCD, and unexplained poor performance, emphasize the importance of studies that enable early detection and risk stratification in high-performance horses. Recent advancements in wearable ECG technology now enable reliable cardiac monitoring during high-intensity exercise as part of regular training and racing.

By leveraging large-scale ECG data collected during intense field exercise sessions, we aimed to improve the understanding of the frequency and nature of exercise-associated arrhythmias, and to provide preliminary insight into their prognostic value for subsequent race performance. Such knowledge is required for evidence-based guidelines for managing equine heart health in racing settings. Our primary objectives were to describe frequencies of various types of arrhythmias that occurred during training gallops and barrier trials (“exercise events”) and explore potential associations among ECG variables measured during exercise events and subsequent race performance. We also assessed the extent of clustering of ECG abnormalities within horses, correlations among ECG variables from different phases of the same exercise event, and among the same ECG variables at consecutive exercise events.

## Materials and methods

### Study design and data collection

#### Overview

A risk-based case–control study was conducted with data collected both retrospectively (before the study was conceptualized) and prospectively. Race starts by Thoroughbred racehorses currently racing at the Hong Kong Jockey Club (HKJC) in which the horse finished in the last 3 (case or poor-performance starts) or finished in the first 3 (control or good-performance starts) of a HKJC-sanctioned race were selected. For each enrolled start, the most recent previous exercise event during which ECG data had been recorded was identified. The ECG variables among these exercise events were described and associations between these ECG variables with outcome in the subsequent race were assessed.

#### Enrollment criteria

Starts were eligible for enrollment if the:

- race was in Hong Kong (at either Sha Tin or Happy Valley racecourse) from May 2021 to July 2022 inclusive (ie, over part or all of each of 2 consecutive racing seasons),
- horse was trained by a specific trainer,
- horse finished either in the last 3 or the first 3 horses in the race, and
- there were previous exercise events for the horse during which an ECG was recorded.

Only starts by horses trained by 1 trainer, selected before the study, were eligible because that trainer frequently used Equimetre technology (Arioneo, France) to record time, speed, stride characteristics (stride length and frequency), heart rate (HR), and ECG data during exercise events. Eligible starts were selected and ECG recordings for previous exercise events back to the horse’s penultimate race start were obtained.

### ECG data analyses

The ECG recordings were analyzed by 2 authors (E.V.E., F.T.W.) blinded to the performance category.

**Figure 1:**
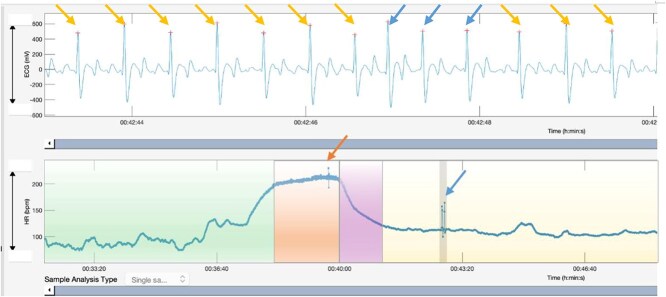
Snapshot of the ECG and HR during a training session recorded with the Equimetre and analyzed in the Kubios software (Kubios HRV Premium 3.5.0). The recording was divided into 4 phases: warm-up (green), maximal phase (orange), fast recovery (purple), and slow recovery (yellow). In this recording, bottom HR trace shows a single ectopic depolarization at the end of the maximal phase (orange arrow) and a triplet during the slow recovery phase (blue arrow). The ECG tracing on the top part of the figure shows the 3 consecutive ectopic depolarizations constituting the triplet identified by the blue arrows; the yellow arrows highlight the normal beats. Abbreviation: HR = heart rate.

**Figure 2:**
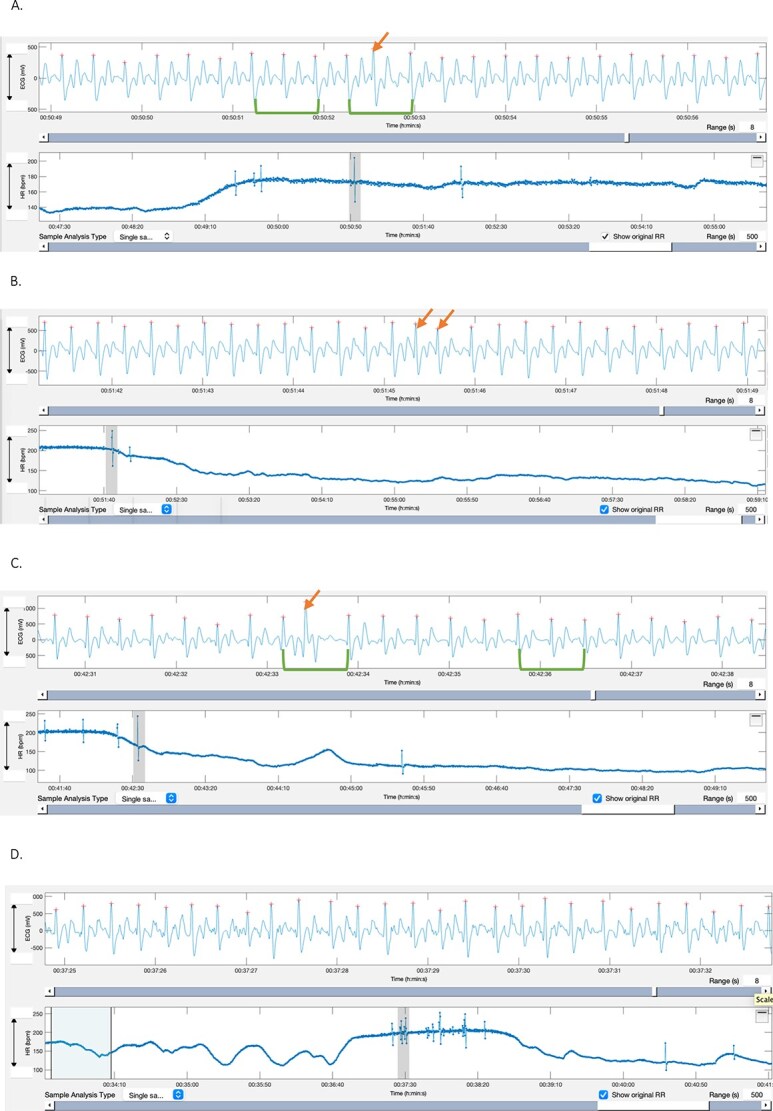
Examples of types of arrhythmia and their characteristics recorded by the Equimetre (Arioneo) as analyzed with the Kubios software (Kubios HRV Premium 3.5.0). (A) Premature narrow complex with a compensatory pause (red arrow). The green calipers indicate the distance between 2 consecutive normal RR intervals. When applied to the 2 beats framing the premature complex, the distance is identical, characterizing a compensatory pause. (B) Couplet: 2 consecutive premature narrow complexes (red arrows). (C) Premature wide complex with a compensatory pause (red arrow). The green caliper indicates the distance between 2 consecutive normal RR intervals. When applied to the 2 beats framing the premature complex, the distance is identical, characterizing a compensatory pause. (D) Bout of paroxysmal atrial fibrillation (pAF). All the RR intervals falling under the orange caliper are irregularly spaced indicating an irregularly irregular rhythm. Green lines represent the RR distance between 2 normal beats and the yellow and orange bars highlight the RR variability. Abbreviation: pAF = paroxysmal atrial fibrillation.

For each exercise event, the recording was divided into 4 phases: warm-up, maximal, and fast and slow recovery, based on the HR curves (Figure 1). Ectopic beats were identified as outliers on the HR curve (Figure 1). A detailed review of the analysis of ECGs and classification of ectopic events is included in Appendix 1. Examples of different types of arrhythmic events are shown in Figure 2.

### Statistical analyses

Methods used for the statistical analyses are detailed in Appendix 1.

## Results

### Numbers of race starts, horses, and ECGs

**Figure 3:**
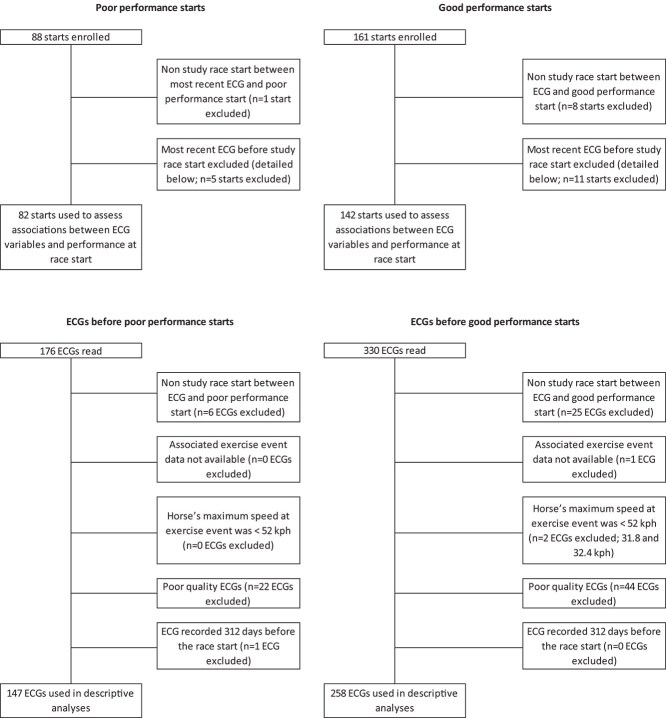
Flow charts showing numbers of starts, ECGs, and exclusions. Overall, 84% (147/176) and 78% (258/330) of ECGs recorded before poor-performance and good-performance race starts, respectively, were used in descriptive analyses.

Eighty-eight poor-performance and 161 good-performance starts from 73 racehorses initially were selected, with 506 ECGs at prior exercise events. After exclusion of starts because of poor-quality ECGs (Figure 3), 82 poor-performance and 142 good-performance race starts from 71 horses were used for analyses of associations with race performance using the most recent ECG before the race start. Of the 506 ECGs, 405 were used in descriptive analyses. These comprised 84% (147/176) and 78% (258/330) of ECGs recorded before poor-performance and good-performance race starts, respectively.

The mean number of ECGs recorded before each race start was 1.8 (range, 1-6) for poor-performance starts and 1.8 (range, 1-5) for good-performance starts. Among the 71 horses included in the final dataset, 35 contributed both poor- and good-performance starts, 16 contributed only poor-performance starts, and 20 contributed only good-performance starts. The mean numbers of poor- and good-performance race starts per horse were 1.2 (range, 0-4) and 2.0 (range, 0-8), respectively.

Most ECGs (89%; 362/405) were obtained during training gallops, with the remaining 11% (43/405) recorded during barrier trials. For each race start, only the final ECG recorded closest to the event was included in analyses of associations with race start performance.

The mean and median durations of the maximal exercise phase were 122 and 120 s, respectively, with 10th-90th percentiles ranging from 85 to 165 s. Mean (range) durations were 125 s (65-210; *n* = 146) for ECGs preceding poor-performance starts and 121 s (60-240; *n* = 244) for ECGs preceding good-performance starts.

### Frequencies of various arrhythmia types

**Figure 4:**
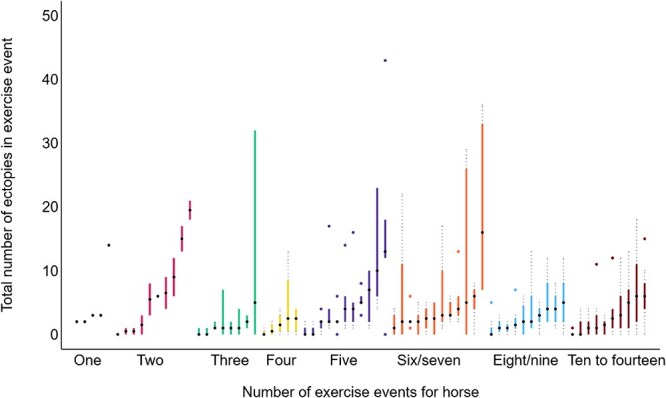
Distributions of total number of ectopic depolarizations for all 4 exercise phases pooled for the exercise event by each horse (*n* = 71 horses). Each box and whisker plot corresponds to an individual horse. Each horse had 1 exercise event (black; left-hand group), or 2 (red) 3, (green), 4 (yellow), 5 (purple), 6 or 7 (orange), 8 or 9 (light blue), or 10-14 (brown) exercise events. Boxes extend from the 25th to the 75th percentiles, and the black dots indicate the medians. Upper whiskers (gray dotted lines) extend to the largest value that is ≤ 75th percentile plus 1.5 times the interquartile range. Lower whiskers extend to the smallest value that is ≥ 25th percentile minus 1.5 times the interquartile range. More extreme values are shown as round markers. Within color groups, horses are in ascending order based on the median of their total numbers of ectopic depolarizations. There was also a value (not plotted) of 81 for the 10th horse in the purple group (horses with 5 exercise events).

Arrhythmias were common in both groups: 73% of ECGs preceding good-performance race starts and 81% of ECGs preceding poor-performance race starts showed at least 1 detectable arrhythmia. Figure 4 presents the total number of ectopic depolarizations detected across all 4 exercise phases for each horse. With the exception of a single horse that contributed 2 ECGs without ectopic beats detected, all horses had at least 1 ectopic depolarization during the maximal phase of at least 1 exercise event.

Distributions of arrhythmia types are summarized in Table 1. When all phases and types of ectopic beat were pooled, 73% of the 258 ECGs preceding good performance and 81% of the 147 ECGs preceding poor performance contained ≥ 1 ectopic beat. Multiple ectopic events were also frequent: 41% of ECGs preceding good performance and 50% of those preceding poor performance had ≥ 3 ectopic depolarizations.

**Table 1:** Distributions of numbers of ectopic depolarizations or, for incidence rates, numbers of ectopic depolarizations per horse-minute of maximal exercise. “Percentage” is the percentage of exercise events (EEs) where the number of ectopic depolarizations were ≥ 1 or ≥ 3.

**Exercise events phase and ectopy type**	**ECGs preceding good-performance race starts** ^**a**^				**ECGs preceding poor-performance race starts** ^**b**^			
	**Percentage ≥ 1**	**Percentage ≥ 3**	**90th percentile**	**Max**	**Percentage ≥ 1**	**Percentage ≥ 3**	**90th percentile**	**Max**
***All 4 phases pooled***								
**All types**	73%	41%	9	36	81%	50%	13	81
***Warm-up phase***								
**All types**	14%	6%	1	17	10%	1%	1	10
***Maximal exercise phase***								
**All types**	57%	25%	5	16	70%	35%	10	41
**All types (incidence rate)**	60%	10%	3	8	70%	16%	5	25
**Wide compensatory**	28%	11%	3	12	39%	17%	3	22
**Wide compensatory (incidence rate)**	29%	5%	2	8	39%	6%	2	15
**Wide noncompensatory**	0%	0%	0	3	5%	3%	0	9
**Wide noncompensatory (incidence rate)**	0%	0%	0	2	5%	1%	0	4
**Narrow compensatory**	24%	5%	2	8	32%	6%	2	14
**Narrow compensatory (incidence rate)**	25%	2%	1	5	32%	1%	1	7
**Narrow noncompensatory**	18%	6%	2	16	21%	13%	3	39
**Narrow noncompensatory (incidence rate)**	19%	2%	1	7	21%	5%	2	23
**Wide^c^**	28%	11%	3	12	39%	20%	5	22
**Wide (incidence rate)^c^**	29%	5%	2	8	40%	7%	2	15
**Narrow^c^**	39%	13%	3	16	46%	19%	5	39
**Narrow (incidence rate)^c^**	41%	4%	2	7	45%	8%	2	23
**Compensatory^d^**	47%	17%	4	12	63%	23%	5	22
**Compensatory (incidence rate)^d^**	50%	7%	2	8	63%	8%	3	15
**Noncompensatory^d^**	19%	7%	2	16	24%	15%	4	39
**Noncompensatory (incidence rate)^d^**	20%	2%	1	7	23%	7%	3	23
**Couplets^d^**	12%	0%	1	6	18%	2%	1	5
**Triplets^d^**	5%	1%	0	3	7%	1%	0	4
**Quadruplets^d^**	2%	0%	0	2	1%	0%	0	1
**5 or more^d^**	1%	0%	0	1	1%	0%	0	1
**Paroxysmal AF**	1%	0%	0	1	4%	0%	0	1
**Triplets and quadruplets pooled^d^**	7%	2%	0	3	7%	1%	0	4
**5 or more and paroxysmal AF pooled**	2%	0%	0	1	5%	0%	0	1
***Fast recovery phase***								
**All types**	28%	9%	2	21	26%	5%	2	8
***Slow recovery phase***								
**All types**	14%	7%	1	24	13%	3%	1	78

^a^Number of ECGs = 258 except for incidence rates where *n* = 244 and for slow recovery from couplets to end of table where *n* = 257.

^b^Number of ECGs = 147 except for incidence rates where *n* = 146.

^c^Compensatory and noncompensatory pooled.

^d^Wide and narrow pooled.

Abbreviation: AF = atrial fibrillation.

Incidence rates during the maximal phase further demonstrated high arrhythmic burden. Among ECGs preceding good performance (*n* = 244), 60% had incidence rates ≥ 1 ectopic beat per horse-minute and 10% had rates ≥ 3 ectopic beats per horse-minute. Corresponding proportions for ECGs preceding poor performance (*n* = 146) were higher, at 70% and 16%, respectively.

Arrhythmias were most prevalent during the maximal phase: 57% of ECGs in the good-performance group and 70% in the poor-performance group had arrhythmic events detected during this phase. Complex patterns included triplets (5% and 7% of ECGs in the good- and poor-performance groups, respectively), quadruplets (2% and 1%), complex arrhythmias (data in Table 1), and paroxysmal atrial fibrillation (pAF) (0.8% and 4.1%).

### Correlations between phases of same exercise events

Estimated coefficients for correlations between the number of ectopic depolarizations occurring during the maximal phase and the number of ectopic depolarizations recorded at each other exercise phase of the same exercise event were nearly 0, regardless of the type of ectopic beat. Correlation coefficients were maximal vs warm-up phase (−0.02; 95% CI, −0.12 to 0.08), maximal vs fast recovery phase (0.07; 95% CI, −0.03 to 0.17), and maximal vs slow recovery phase (−0.01; 95% CI, −0.10 to 0.09).

### Time of onset of ectopic depolarizations during exercise events

**Figure 5:**
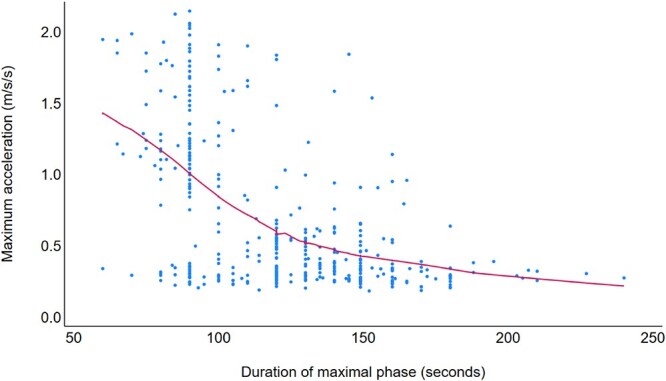
Scatterplot of maximum acceleration by maximal phase duration for 378 exercise events. The regression line is the locally weighted regression (lowess) line (red). Both maximum acceleration and maximal phase duration were available for 378 of the 405 exercise events.

Of the 249 exercise events with ectopic beats detected during the maximal phase, the relative time of onset (beginning, middle, or late in that phase) was available for 233 exercise events. Times of onset varied with exercise duration: ectopies generally appeared earlier in shorter events (<120 s, median maximal phase duration) and later in longer events (≥120 s). In shorter events (*n* = 106), ectopy began at the beginning in 40% (42/106) and in the middle in 44% (47/106). In longer events (*n* = 127), ectopic beats began at the beginning in 25% (32/127) and the middle in 56% (71/127). Maximum acceleration was, on average, higher in events with shorter maximal phases (Figure 5).

### Clustering of arrhythmias by horse

The number of ectopic depolarizations of any type during the maximal phase showed moderate clustering by horse (Table 2). This clustering did not appear to be driven by consistent differences in exercise intensity among horses, because intraclass correlation coefficients estimates adjusted for maximum heart rate and maximum speed were similar to unadjusted estimates (results not shown).

**Table 2:** Intraclass correlation coefficients for selected ECG variables by horse (Nb. = number).

**ECG variable**	**Intraclass correlation coefficient estimate**	**95% CI**
**Ectopic depolarizations in any phase (≥1 or none)**	0.24	0.12-0.42
**Ectopic depolarizations in maximal phase (≥1 or none)**	0.25	0.13-0.42
**Nb. ectopic depolarizations (all types; all phases pooled)**	0.32	0.21-0.45
**Nb. ectopic depolarizations (all types; maximal phase)**	0.39	0.27-0.53
**Nb. wide compensatory ectopic depolarizations (maximal phase)**	0.12	0.05-0.24
**Nb. narrow compensatory ectopic depolarizations (maximal phase)**	0.15	0.05-0.43
**Nb. wide ectopic depolarizations (maximal phase)^a^**	0.12	0.05-0.25
**Nb. narrow ectopic depolarizations (maximal phase)^a^**	0.36	0.24-0.50
**Nb. compensatory ectopic depolarizations (maximal phase)^b^**	0.21	0.10-0.37
**Nb. ectopic depolarizations (all types; fast recovery phase)**	0.13	0.07-0.24
**Nb. narrow ectopic depolarizations (fast recovery phase)^a^**	0.13	0.06-0.23

^a^Compensatory and non compensatory pooled.

^b^Wide and narrow pooled.

### Correlations in number of ectopic depolarizations between exercise events before the same race start

**Figure 6:**
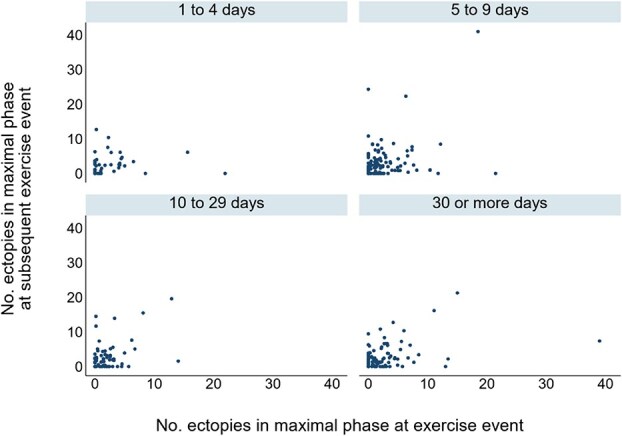
Scatter graphs of number of ectopic depolarizations occurring during the maximal phase at an exercise event and the corresponding number at the next exercise event where the interval to the next event was 1-4, 5-9, 10-29, and ≥ 30 days later. Markers have been jittered (ie, randomly spread slightly) to reduce overlap.

The number of ectopic depolarizations during the maximal phase was not correlated for exercise events 1-4 days apart (*r* = −0.02; 95% CI, −0.32 to 0.28; *n* = 42 pairs), but showed weak positive correlations for events 5-9 days apart (*r* = 0.40; 95% CI, 0.24-0.54; *n* = 124), 10-29 days apart (*r* = 0.43; 95% CI, 0.22-0.61; *n* = 71), and ≥ 30 days apart (*r* = 0.42; 95% CI, 0.24-0.57; *n* = 97; Figure 6).

**Figure 7:**
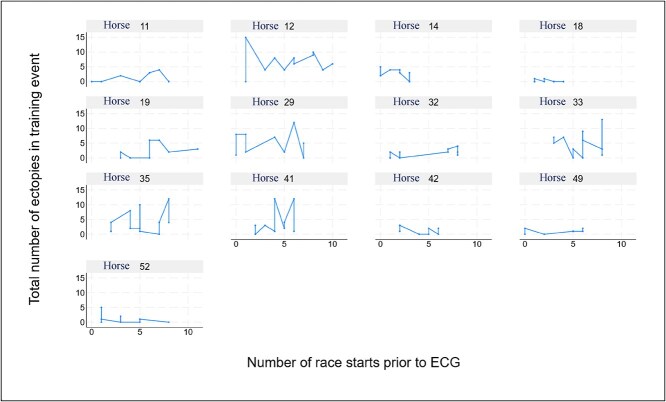
Ectopic beat counts across exercise events plotted against cumulative race starts in horses with ≥ 8 ECG recordings. No consistent temporal trend was observed.

Longitudinal trends in total counts of ectopic beats were also assessed for horses with ≥ 8 ECG recordings, plotted against cumulative race starts from August 15, 2021 (post-layoff period; Figure 7). No consistent temporal trend was observed for ectopic burden or performance (data not shown).

### Associations between number of ectopic depolarizations and subsequent race performance

Mean (range) times from the most recent ECG to race start were 6.4^2-20^ and 6.3^2-21^ days for poor- and good-performance starts, respectively.

Associations were evaluated for the same 11 ECG variables used in the clustering analysis (binary: no ectopic beat vs ≥ 1 ectopic beat) across all exercise phases pooled and for the maximal phase, and for 9 selected ECG count variables. Fractional polynomial regression of continuous ECG counts provided no evidence of nonlinear relationships with subsequent race performance, supporting the use of linear models for all continuous variables. Results are summarized in Table 3.

**Table 3:** Associations between selected ECG variables from the most recent ECG before a race start and finishing position category (poor performance and good performance, *n* = 82 and 142, respectively).

**ECG variable**	**Adjusted odds ratio estimate**	**95% CI**	**Likelihood ratio test *P*-value**
**Ectopic depolarizations in any phase (≥1 or none)**	1.68^a^	0.71-3.97	.236
**Ectopic depolarizations in maximal phase (≥1 or none)**	1.81^a^	0.86-3.81	.021
**No. ectopic depolarizations (all types; all phases pooled)**	1.04^b^	0.98-1.10	.095
**No. ectopic depolarizations (all types; maximal phase)**	1.15^b^	1.04-1.28	.008
**No. wide compensatory ectopic depolarizations (maximal phase)**	1.16^b^	0.98-1.37	.054
**No. narrow compensatory ectopic depolarizations (maximal phase)**	1.03^b^	0.83-1.28	.185
**No. wide ectopic depolarizations (maximal phase)^c^**	1.19^b^	1.01-1.41	.026
**No. narrow ectopic depolarizations (maximal phase)^c^**	1.12^b^	0.98-1.27	.046
**No. compensatory ectopic depolarizations (maximal phase)^d^**	1.11^b^	0.97-1.27	.063
**No. ectopic depolarizations (all types; fast recovery phase)**	0.81^b^	0.61-1.08	.088
**No. narrow ectopic depolarizations (fast recovery phase)^c^**	0.84^b^	0.61-1.15	.115

^a^Estimated odds of poor performance at starts where most recent ECG had ≥ 1 ectopic depolarizations relative to starts where most recent ECG had none. Odds ratio estimates are adjusted for analyst.

^b^Estimated odds of poor performance for each additional 1 ectopy; see also Figure 5. Odds ratio estimates are adjusted for analyst.

^c^Compensatory and noncompensatory pooled.

^d^Wide and narrow pooled.

**Figure 8:**
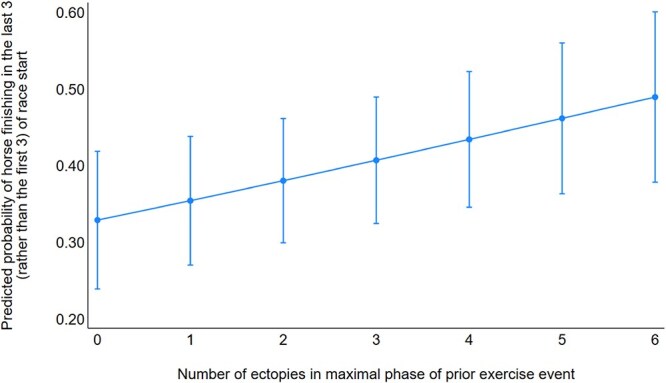
Predicted probabilities of horse finishing in the last 3 (rather than the first 3) at a race start by number of ectopic depolarizations in the maximal phase of the ECG at the most recent exercise event prior to that race start. 0 and 6 ectopic depolarizations were, respectively, the 10th and 90th percentiles for the most recent ECGs before race starts. Error bars indicate 95% CI of predicted probabilities.

For each additional ectopic beat recorded during the maximal phase, the odds of finishing in the last 3 positions (vs first 3) in the next race start increased by a factor of 1.15 (95% CI, 1.04-1.28; Figure 8). Wide morphology ectopic beats were also associated with worse performance (OR, 1.19; 95% CI, 1.01-1.41; *P* = .026).

Complex arrhythmic patterns were infrequent overall but occurred more often in ECGs preceding poor-performance race starts. Paroxysmal atrial fibrillation was identified in 11 of the 224 ECGs recorded immediately before a race start. Of these, 7 ECGs preceded poor-performance starts and 4 preceded good-performance starts. The interval between the exercise event during which pAF was detected and the subsequent race start ranged from 6 to 20 days for poor-performance starts (6, 7, 8, 8, 10, 11, and 20 days) and from 4 to 12 days for good-performance starts (4, 6, 11, and 12 days).

Other complex arrhythmias, including triplets, quadruplets, and runs of 5 or more consecutive ectopic depolarizations, were uncommon but were observed more frequently during the maximal exercise phase and were numerically more prevalent in ECGs preceding poor-performance than good-performance starts (Table 1). Owing to the low number of events, formal statistical analyses of associations between complex arrhythmias and subsequent race performance were not performed.

## Discussion

We have described the frequencies, type, and incidence rates of cardiac arrhythmias in a large cohort of racehorses during intense exercise under field conditions and have evaluated associations between arrhythmic patterns and subsequent race performance. Our results indicate that ectopic beats are common in this population of fit racehorses, occur predominantly during maximal exercise, and are strongly associated with performance during the subsequent race. Importantly, horses classified as poor performers had a higher frequency, incidence rate, and complexity of arrhythmias compared with good performers. These findings highlight an important association between arrhythmia burden and suboptimal race performance that has not been demonstrated previously. The association of ectopic beats with EASCD was not examined in our study, but merits consideration.

This study was enabled by advances in technology that allow collection of qualitative ECGs during maximal exercise under field conditions, technology that is now widely available. Recordings were obtained during training rather than racing, due to regulatory constraints, although barrier trials likely provide a viable proxy for race-day effort. Consistent with this approach, horses with EASCD are more likely to experience a fatal event during training than racing, emphasizing the importance of monitoring cardiac rhythm under standardized high-intensity exercise conditions before competition.^2^

Although isolated arrhythmias generally are considered benign, our results suggest that such might not always be the case, and that the clinical importance of arrhythmias is dependent on the context, type, and complexity of ectopic patterns. The incidence rate emerged as a useful metric, quantifying the temporal density of ectopic beats during exercise. A higher count during maximal exercise was associated with a higher likelihood of poor performance, indicating that arrhythmia burden and not just presence was negatively associated with race performance. Horses with complex arrhythmias or episodes of pAF could represent the more severe end of this spectrum, but numbers affected were too low to allow formal analyses.

The risk factors for development of ectopic beats were not examined in our study and thus the role, if any, of intrinsic cardiac factors, management (eg, feeding, supplements, and electrolyte administration), or comorbidities (eg, asthma, gastric ulcers, and musculoskeletal lesions) could not be assessed.

Interpretation of narrow vs wide complexes in our study must be made with caution, given the single lead nature of the recordings and the decreased morphological distinction inherent to the display. In principle, narrow ectopic complexes are most consistent with supraventricular premature depolarizations (atrial or junctional), whereas wide complexes likely reflect either ventricular ectopy or supraventricular beats conducted with aberrancy. Because only 1 vector was available, definitive classification of supraventricular vs ventricular ectopia was not possible. Nevertheless, the finding that ectopic beats with wide morphology were associated with higher odds of poor performance suggests that ectopic beats with more severe conduction disturbance or delayed activation could carry stronger physiological or performance implications than morphologically narrow ectopic beats. This distinction, although not a definitive diagnosis of origin of ectopic beats, provides clinically relevant information for practitioners. If these beats typically originate from the ventricular myocardium, findings support the hypothesis that ventricular arrhythmias impair cardiac output and are clinically relevant when assessing fitness to perform.

Albeit rare overall, complex arrhythmias were more frequent in the poor performance compared with the good-performance group. Episodes of pAF were transient, occurred exclusively during peak exertion, and did not persist into recovery, consistent with previous reports describing short-lasting, exercise-induced atrial fibrillation in racehorses (ter Woort et al., in press). Although the small number of affected horses precluded formal statistical analysis, the overrepresentation of complex arrhythmias and pAF in the poor-performance group suggests that these patterns may represent the more severe end of the arrhythmic spectrum observed during maximal exercise.

The presence of complex arrhythmias during intense exertion may reflect either an underlying arrhythmogenic substrate or a maladaptive electrophysiological response during an exacerbated state of physiological stress. Potential mechanisms include exercise-induced atrial or ventricular stretch, altered autonomic balance, transient myocardial hypoxia, or conduction heterogeneity associated with athletic cardiac remodeling. Complex ventricular arrhythmias and atrial fibrillation previously have been associated with EASCD in racehorses, emphasizing their potential clinical relevance even when episodes are brief and self-terminating. However, in the absence of further clinical diagnostic evaluation, the clinical relevance of these findings in the present cohort remains speculative.

No correlation was observed between ECG abnormalities at peak exercise and those recorded during warm-up or recovery, indicating that it is not possible to predict the frequency or characteristics of ectopic beats during maximal exercise by what is observed during warm-up or recovery. The frequency of arrhythmias during these submaximal phases did not differ significantly between performance groups. We found the frequency of arrhythmias to be markedly higher during the maximal exercise phase, compared with warm-up or recovery, which aligns with studies in Thoroughbred, Standardbred, and Chuckwagon racehorses but contrasts with other studies that reported higher prevalence of arrhythmias either during warm-up^7^ or post-exercise recovery phases^6,10,11^ in horses. Our results emphasize the diagnostic importance of ECG monitoring during maximal exertion in Thoroughbred racehorses.

The occurrence of arrhythmias at an earlier stage during shorter exercise events might suggest that the higher physiologic stress of a steeper acceleration to reach peak speed could trigger arrhythmic events, although the pathophysiologic basis for this possibility was not investigated during our study.

Myocardial remodeling and intermittent hypoxia are well-documented in athletic horses, especially those undergoing sustained high-intensity training.^12^ Although such physiological adaptations are often compensatory, they can alter myocardial structure and conduction pathways, providing an arrhythmogenic substrate.^13,14^ Both complex ventricular arrhythmias and atrial fibrillation are linked to EASCD in racehorses.^2,9,15-18^ Our findings emphasize the potential relevance of monitoring ECGs during high-intensity exercise as a preventive strategy. As such, routine cardiac screening, particularly during peak exertion, could be incorporated into preparticipation evaluations to identify horses at higher risk. Electrocardiograms recorded during training mirror those recorded during racing in Chuckwagon racehorses, supporting their use as a surrogate for race day monitoring.^19^ Studies in human athletes advocate for exercise ECGs as part of cardiovascular risk assessments,^20-22^ and similar approaches could be valuable in equine medicine.

The presence and frequency of ectopic beats during maximal exertion correlated with worse race performance. Horses with higher ectopy counts were more likely to finish in the bottom 3 positions (OR, 1.15; *P* = .01), and those with any ectopy were nearly twice as likely to underperform (OR, 1.8; *P* = .02). Although the causal relationship remains uncertain, these findings offer valuable predictive insight. Factors such as pain, poor conditioning, or subclinical disease predispose horses to both ectopy and poor outcomes, confounding the observed relationship between ectopy and poor performance.^1,15,23^ Still, the predictive value of training ECGs provides a practical opportunity for performance management and injury prevention.

The absence of a consistent ECG pattern during longitudinal follow-up in several racehorses suggests that ectopic activity is variable and does not consistently correlate with changes in fitness or exposure to intense exercise and racing. This variability emphasizes the complexity of interpreting ECG findings based on single recordings in athletic horses and the importance of repeated assessments and contextual interpretation when evaluating arrhythmias in relation to performance.

Our study had some limitations. The ECG signal obtained from different devices can differ in appearance. Although the Equimetre device records at 500 Hz, preserving the frequency content of the QRS complex, the displayed morphology is influenced by the single-vector lead configuration and by amplitude and time-scaling applied during Kubios visualization. These factors can make beat-to-beat morphological differences appear less pronounced than on a conventional multi-lead surface ECG. Consequently, interpretation of QRS morphology, particularly the distinction between narrow and wide complexes should be made with this limitation in mind. Reliance on a single-lead ECG also could have resulted in undetected ectopic activity and did limit the classification of arrhythmias, as in other studies (eg, distinction between supraventricular or ventricular ectopy). Although a number of recordings were double-reviewed, a formal interobserver reliability assessment was done in a previous validation study.^23^

Recordings were obtained during training, not racing, because of regulatory constraints although barrier trials likely provide a viable proxy for race day effort. Consistent with this approach, horses experiencing sudden cardiac death are more likely to do so during training than during racing, emphasizing the importance of monitoring cardiac rhythm under standardized high-intensity exercise conditions before competition.^2^

All horses were from a single training stable, ensuring protocol consistency but potentially limiting generalizability. Finally, no clinical data were available to evaluate underlying cardiac conditions or comorbidities, preventing full contextualization of the arrhythmic findings.

## Conclusion

Cardiac arrhythmias during maximal exertion are common among Thoroughbred racehorses in Hong Kong and are more frequent in horses with subsequent poor race performance. The profile of their timing, frequency, and complexity, especially during high-intensity exercise, likely has prognostic value with regard to subsequent race performance. Although causality was not established, wearable ECG monitoring during training could serve as a powerful tool for early detection of abnormalities, performance optimization, and injury prevention. Incorporating wearable ECG monitoring into training programs would enhance the early detection of at-risk individuals, contributing to better-informed clinical decisions and improved health, welfare, and performance outcomes for equine athletes.

## Off-label antimicrobial declaration

The authors declare no off-label use of antimicrobials.

## Institutional animal care and use committee or other approval declaration

The authors declare no institutional animal care and use committee or other approval was needed.

## Appendix 1 Material and methods supplementary information

### ECG data analyses

The ECG recordings were analyzed by two authors (EVE, FTW) using Kubios software (Kubios HRV Premium 3.5.0) with each ECG assessed by one person except for ECGs that were read by each author, in which case ECGs from one reading were selected randomly for use in the study. Analysts were not aware of whether the associated subsequent race start was a poor or good performance start. Each ECG recording was classified as excellent, good or poor diagnostic quality. Any recording that had > 5% of noise during the maximal phase of exercise was classified as being of poor diagnostic quality and excluded from further analysis.

For each exercise event, the recording was segmented into 4 phases: warm-up, maximal and fast and slow recovery, based on the HR curves (Figure 1). The warm-up phase began at the start of the recording and included the period of acceleration to the maximal phase. The maximal phase was defined as the phase in which heart rates reached a steady state > 185 bpm after an acceleration phase and ended when there was an abrupt decrease in HR. The fast recovery phase corresponded to the rapid decrease in heart rate after the maximal phase and ended when an inflection in the HR curve occurred; the slow recovery phase began at the end of the fast recovery, when heart rate was consistently < 120 bpm, and continued until the end of the recording.

Ectopic beats were identified as outliers on the HR curve (Figure 1), after which the durations of the corresponding RR and RS intervals were measured using dedicated caliper software (Caliper DV®). A variation in the RR interval > 4% was defined as a premature depolarization (ectopic beat).

An ectopic beat was classified as narrow if its duration and morphology were similar to adjacent normal beats. It was classified as wide if the QRS complex appeared visually broader, of clearly different morphology, including increased apparent duration, altered polarity, or noticeably different amplitude, or both. Differentiation between narrow and wide complexes was therefore based primarily on subjective visual assessment, with caliper measurements used only when uncertainty remained.

For each ectopic beat, the subsequent proximate pause was classified as either compensatory (CP) if the distance between the two normal beats with the ectopic beat in between (normal-ectopic-normal) was the same as twice the normal RR interval or non-compensatory (NCP) if the distance between the two normal beats with the ectopic beat in the middle (normal-ectopic-normal) is less than twice the normal RR interval.

For each exercise event, the number sof each type of ectopic beat were counted and classified as narrow compensatory, narrow non-compensatory, wide compensatory, or wide non-compensatory. Occurrences of couplets, triplets, quadruplets, and more complex arrhythmias were documented. An arrhythmia was defined as complex if it consisted of a succession of ≥5 abnormal depolarizations, regardless of morphology. Paroxysmal atrial fibrillation (pAF) was defined as a continuous sequence of ≥6 irregularly irregular RR intervals with QRS complexes of comparable narrow morphology, resolving spontaneously during recovery. Because only a single-lead ECG was available, episodes classified as pAF could not be definitively differentiated from runs of multifocal atrial ectopic depolarizations. Examples of different types of arrhythmic events are shown in Figure 2.

For each exercise event, the duration of each phase was recorded and, for each phase, numbers of each type of ectopic beat were recorded. The onset of ectopic depolarizations during the maximal phase was further documented as being at the beginning, middle, or end of the maximal phase of the exercise event if they occurred in the first, middle or last third of the maximal phase, respectively. The total number of ectopic depolarizations for the entire exercise event was the sum of the numbers of ectopic depolarizations in each of the 4 phases.

Incidence rates (number of ectopic depolarizations per horse-minute of maximum phase exercise) for each exercise event were calculated for each ectopic beat type as the quotient of number of occurrences of that type of ectopic beat and the duration of the maximal phase.

### Statistical analyses

Statistical analyses were performed using Stata (version 18, StataCorp, College Station, Texas, USA). Correlations between continuous ECG variables were assessed using Pearson’s correlation coefficients, with 95% CI calculated using Fisher’s transformation. Stata’s -ci2- command was used for analyses.

In preliminary analyses, there was no evidence of positive association between maximal phase duration and numbers of ectopic depolarizations of various types, and thus absolute numbers of ectopic depolarizations were analyzed along with incidence rates of various ectopic beat types in the maximal phase.

Clustering of ECG variables within individual horses was described using intraclass correlation coefficients (ICC). For ECG variables analyzed as continuous data, multilevel mixed-effects linear regression models were fitted using Stata’s -mixed- command in which ECG was nested within horse with horse fitted as a random intercept, and ICC were calculated using Stata’s -estat icc- command as*:*

\begin{eqnarray*}
\text{Horse level intercept variance total variance}
\end{eqnarray*}

The δ method was used to estimate the standard errors of the ICC, and the logit transformation was used to obtain CIs.

For ECG variables analyzed as binary data, multilevel mixed effects logistic regression models were fitted using Stata’s -melogit- command, and ICC were calculated as:

\begin{eqnarray*}
\text{ICC = Horse level intercept}/\left(\mathrm{Horse\ level\ intercept\ variance + \pi{^2/3}}\right)
\end{eqnarray*}

Intraclass correlation coefficients were calculated both unconditionally and as residual ICC, the latter by fitting maximum HR and maximum speed during the exercise event as fixed effect covariates.

Intraclass correlation coefficients were estimated for the binary variables, ≥1 or 0 ectopic depolarizations, for all exercise phases pooled and separately for the maximal phase, and for the 9 ECG count variables where a modest or high proportion of counts were > 0 (specifically where ≥20% of both ECGs preceding poor performance and good performance race starts were > 0). These 9 ECG variables were selected because for these variables there was adequate variation in the data for meaningful analyses.

Associations between ECG variables from the most recent ECG before the race start and finishing position category (poor performance or good performance) at that start were evaluated using multilevel logistic regression models fitted using Stata’s -melogit- command, with analyst fitted as a categorical variable as a fixed effect and horse fitted as a random intercept. Analyst was fitted to remove any confounding caused by differences in reading ECGs between analysts. For continuous ECG variables, linearity in the logit was first assessed using 1- and 2-dimension fractional polynomials, fitted using Stata’s -fp- command.

The usefulness of number of ectopic depolarizations occurring during the maximal phase at an exercise event in predicting that number during the maximal phase at the next exercise event was explored by assessing correlations among numbers at consecutive exercise events, where those existed for individual horses.

## Abbreviations

EASCD: exercise-associated sudden cardiac death
HKJC: Hong Kong Jockey Club
pAF: paroxysmal atrial fibrillation
